# Amyotrophy in Syringomyelia

**Published:** 1908-12-12

**Authors:** 


### NEUROLOGY.

### AMYOTROPHY IN SYRINGOMYELIA.

It is a fact that receives less recognition than it should that one of the most prominent symptoms of syringomyelia may be muscular atrophy. The disease may consequently simulate amyotrophic lateral sclerosis or progressive muscular atrophy. That this should be so is not surprising when one remembers that the lesion in the cord consists of overgrowth of the substantia gelatinosa around the central canal, together with more or less over-distension of the latter. If these changes extend as far as the anterior horns of grey matter it is clear that there will be destruction of anterior cornual cells; the consequence of this destruction will be atrophy of the corresponding muscles, and reaction of degeneration in them. If the over-distension of the central canal destroys anterior cornual cells in the cervical enlargement, progressive muscular atrophy will be simulated; if in the dorsal region the amyotrophy may escape detection; if in the lumbar enlargement, peripheral neuritis will be simulated, and so on. It might be thought that the diagnosis would be rendered obvious at once by there being inability to distinguish heat from cold and pain from touch in the parts affected by amyotrophy. This is not necessarily so. The atrophy may be pronounced in the hands and arms, for example, whilst the phenomena of dissociated sensations are to be made out only in some other part of the body. In every case of obscure amyotrophy, therefore, it is essential to test not only cutaneous sensibility but also sensibility to heat, cold, and pain, both in the obviously affected regions and in the rest of the body too.

The following is an instance in point. The patient was a young man of 22 who exhibited a classical condition of *main en griffe*. He stated that he was perfectly well up to the age of 20, when he began to notice that his hands were becoming less powerful than they had been, and that they also felt cold and numb. These symptoms had come on apparently without cause and they slowly but steadily advanced. There was no family history of any similar complaint.

At first sight the condition seemed to be that of progressive muscular atrophy, notwithstanding the unusually early age at which it had set in. In favour of this diagnosis could be urged that there were no cerebral symptoms, no visceral troubles, that there was no demonstrable sensory disturbance in the hands or arms notwithstanding their being the site of subjective sensations of coldness and numbness, that the atrophy was limited to the small muscles of the thenar and hypothenar eminences and of the interosseous spaces, that the humero-scapular and the facial muscles were perfectly normal, and that there was nothing in the patient's family history to suggest a primary muscular dystrophy.

On further examination the knee-jerks were found to be exaggerated, and the plantar reflex was extensor upon both sides. This suggested amyotrophic lateral sclerosis; but when a careful investigation of the patient's sensory phenomena was made it was discovered that whereas these were perfectly normal over the greater part of the body, and over both upper and both lower limbs, there was a well-defined and broad band of dissociated anæsthesia varying from 2½ to 4½ inches in vertical extent and reaching, roughly speaking, round the left side of the body from the spine behind to the umbilicus in front. Here the patient could feel quite well in the ordinary sense, but was unable to distinguish heat from cold, or pain from touch.

The diagnosis could not be confirmed by examination of the spinal cord, but syringomyelia accounts for all the symptoms better than any other diagnosis. The possibility of amyotrophy being due to syringomyelia should always be borne in mind.

